# Using the surgical guide for impression-free digital bite registration in the edentulous maxilla—a technical note

**DOI:** 10.1186/s40729-019-0172-8

**Published:** 2019-05-22

**Authors:** George Michelinakis, Dimitrios Nikolidakis

**Affiliations:** 1 Crete Implants Private Dental Practice, 5 Riga Feraiou Sqr, 71201 Heraklion, Crete Greece; 2 ClinicPerio Private Dental Practice, Viannou 1, 71201 Heraklion, Crete Greece

**Keywords:** Intraoral scanning, Bite registration, Digital workflow, Guided implant placement

## Abstract

Studies reporting on the application of digital bite registration for fully edentulous patients rehabilitated with dental implants are scarce. This article describes a technique for intraoral digital registration of the occlusal vertical dimension in a fully edentulous maxilla to be rehabilitated with a fixed implant prosthesis. Following fully guided placement of six maxillary implants, the surgical stent duplicating the existing upper full denture was securely fixed on two anterior implants and sectioned along the center line of the hard palate. An intraoral scanner was used for the digital impression of the maxilla and dentate mandible. The occlusal vertical dimension was registered on each side using the contralateral part of the surgical guide along with the scanbodies on each side. The procedure allowed for the precise digital mounting of the digitized jaws. The maxilla was restored with a full-arch implant-supported prosthesis.

## Background

Accurate impression making is a cornerstone for well-fitting tooth and implant-supported restorations. Impressions can be made with either a conventional or a digital approach. The conventional impression workflow has limitations regarding its accuracy that mainly involves material shrinkage after setting [1], but it is still considered as the gold standard.

The introduction of intraoral scanning (IOS) in recent years has allowed dentists to acquire data directly from the mouth of the patients without the need for a conventional impression material and technique [2, 3]. The accuracy of digital impressions produced with an intraoral scanner has been studied extensively, both for single teeth and short span distances [4–7] as well as long span distances [8–12] with very favorable results compared to the accuracy of conventional impression workflows [13]. Research has recently shifted its focus on the accuracy of intraoral scanning in cases of fully edentulous arches [14–16]. Literature suggests that the accuracy of this technique increases as the number of installed implants increases and the distance between implants decreases [15]. Implant angulation has not been found to play a significant role in digital impression accuracy [16], but implant placement depth is reported to affect accuracy. The visible portion of the scanbody is crucial for the correct registration of the implant position [17].

Another challenge in restoring these patients, either with complete removable or with implant-supported fixed prostheses, is the precise digital registration of the moveable soft tissues [18] and the precise digital registration of the vertical dimension of occlusion [19]. Digitally capturing the bite at the correct occlusal vertical dimension (OVD) with the aid of full-arch fixed implant-retained interim prosthesis as a guide has been previously reported in the literature [20, 21]. For a removable complete denture to serve as a bite registration aid though, it has to maintain adequate intraoral stability. A technique for digital recording of the OVD in a fully edentulous maxilla was recently published [22]. The authors used a silicone index made from putty material at the established OVD as an occlusal rim. The anterior portion of the index between the first premolar areas was cut out to allow for adequate soft tissue scanning without decreasing the stability of the index. Any minor movements of the putty rim were considered acceptable as the maxilla was to be restored with a conventional complete denture.

Restoring an edentulous jaw with a fixed implant-supported prosthesis, on the other hand, requires a more accurate OVD registration. In a recent pilot clinical study, Hassan et al. [23] described a technique whereby a relined duplicate of the patient’s maxillary complete denture was used as a basis for obtaining a precise digital intraoral record. This technique has also been reported previously in obtaining interocclusal records both for CAD-CAM fabrication of an implant placement surgical stent [24] and also for the OVD registration when fabricating the definitive implant-supported prosthesis [25].

Digital bite registration using the Trios IOS has been proven to be accurate in a partially dentate implant scenario, but evidence on edentulous full-arch bite accuracy is lacking [26]. Further research is needed to establish the accuracy of the digital bite registration process in fully edentulous patients where data for alignment is missing.

The purpose of this article is to present an analog procedure that will aid in the digital process of registering the occlusal vertical dimension using an intraoral scanner in a fully edentulous maxilla with six implants.

## Case presentation

**Fig. 1:**
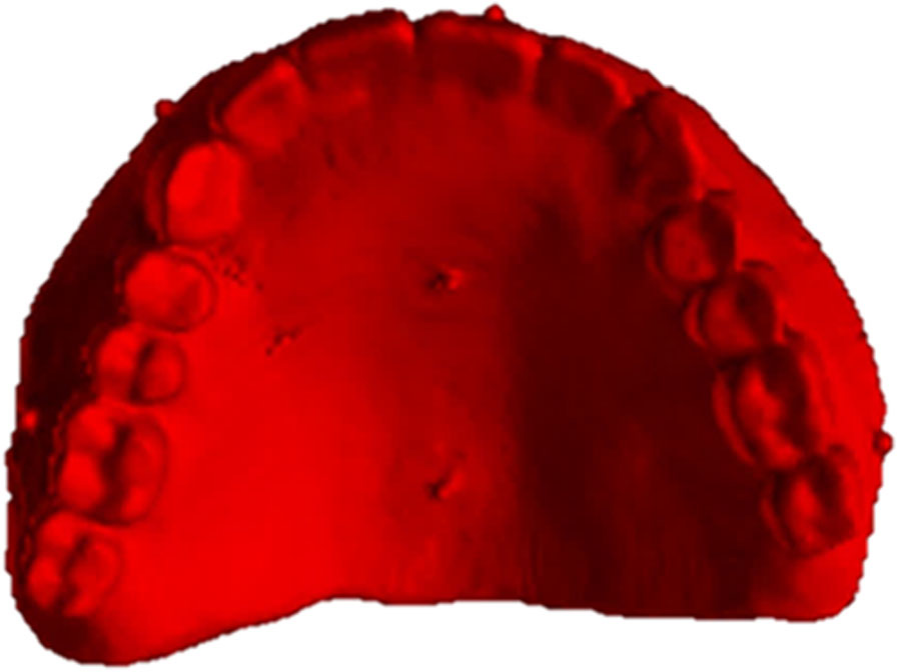
CBCT scan of existing denture

**Fig. 2:**
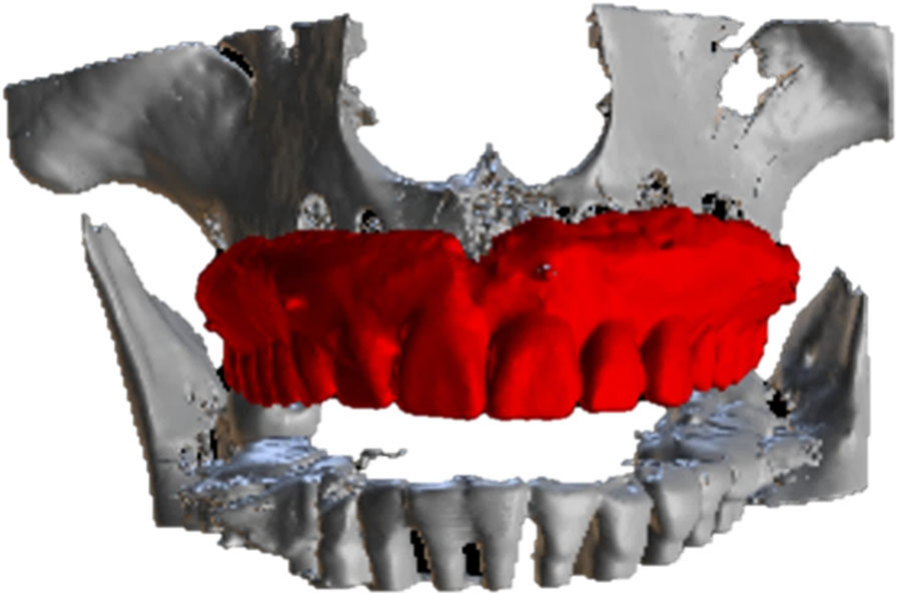
Alignment of denture scan to patient’s maxillary CBCT scan

**Fig. 3:**
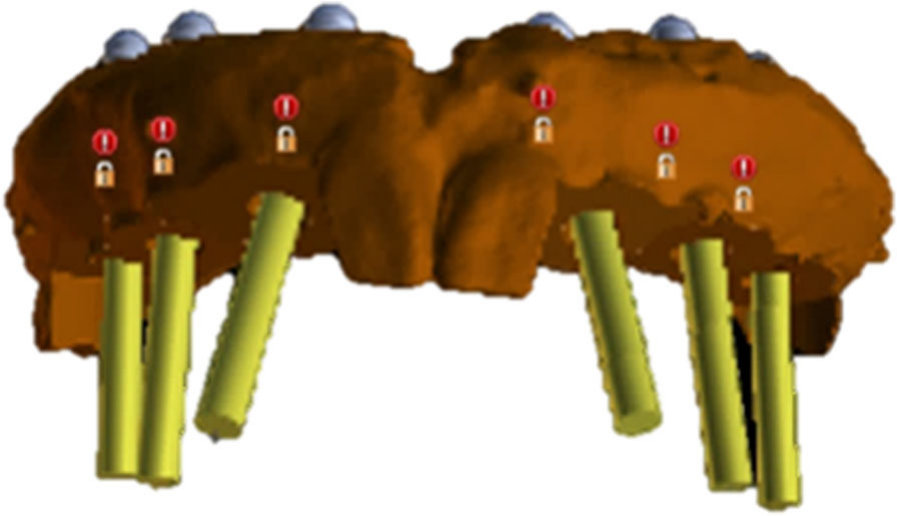
Implant placement simulation and surgical guide design

**Fig. 4:**
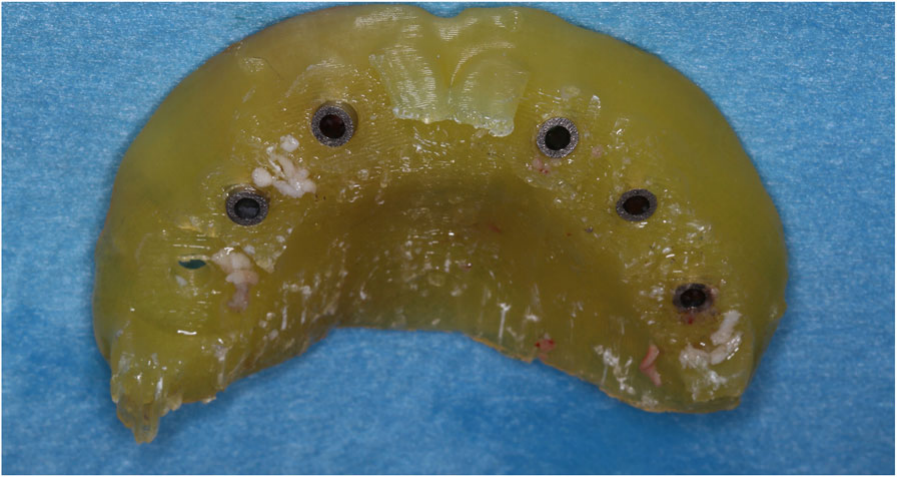
3D-printed surgical guide

A 44-year-old male patient, described in this report, had previously undergone extraction of all upper teeth and was restored with an immediate full denture at the correct OVD and occlusal relation. His mandible was fully dentate, and all the remaining teeth had undergone non-surgical periodontal treatment and were periodontally stable. Following the appropriate healing period, a decision to restore the maxilla with a full-arch fixed prosthesis on six implants was taken. Radiolucent cone beam markers (Blue Sky Bio, USA) were attached to the patient’s existing complete upper denture which was then scanned with a ProMax 3D Mid CBCT appliance (Planmeca Inc., Finland). The patient was also scanned in the same CBCT appliance with the denture in situ. The denture scan was aligned to the maxillary CBCT scan (Figs. 1 and 2) in Blue Sky Plan (Blue Sky Bio, USA) to allow for prosthetically driven implant planning. Implant placement simulation was carried out in the software, and a pilot-guide surgical stent was designed (Fig. 3) and printed in a desktop 3D printer (Lulzbot Mini, USA) using PolyLite PLA material (Polymaker, Netherlands). In order to facilitate correct implant placement, all teeth were removed from the surgical guide during the design process with the exception of the two central incisors and the two first molars (Fig. 4). These four teeth were strategically kept to maintain the established OVD at closure.

**Fig. 5:**
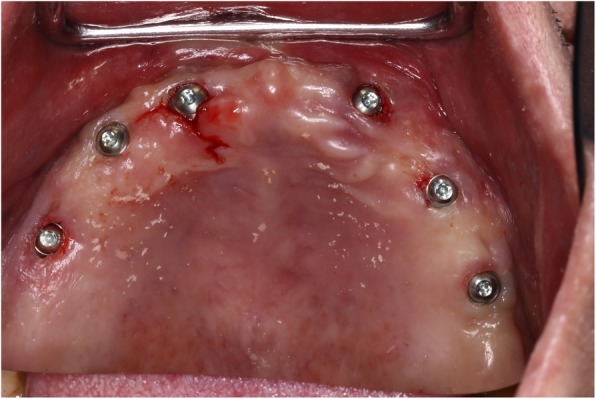
Flapless placement of six maxillary implants

**Fig. 6:**
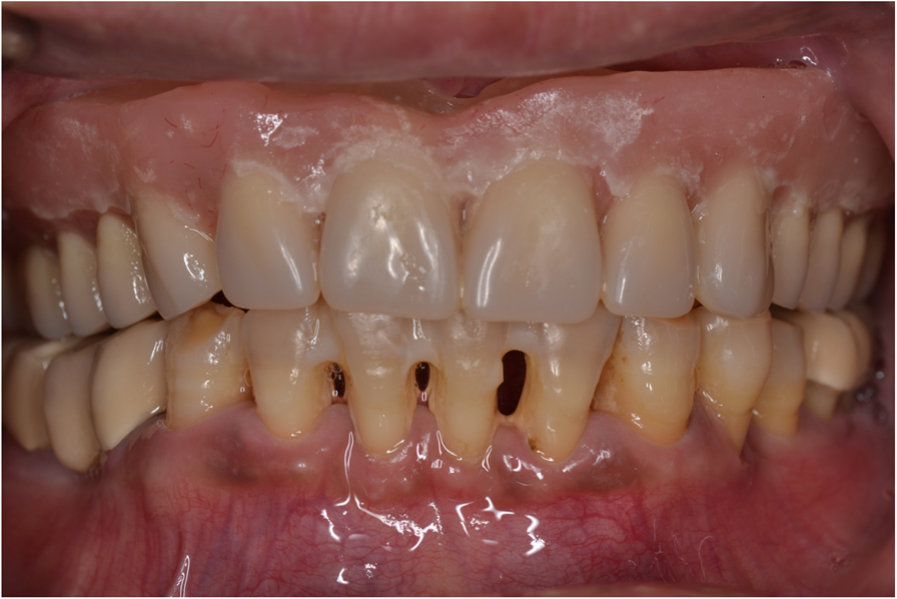
Denture relined with Viscogel

**Fig. 7:**
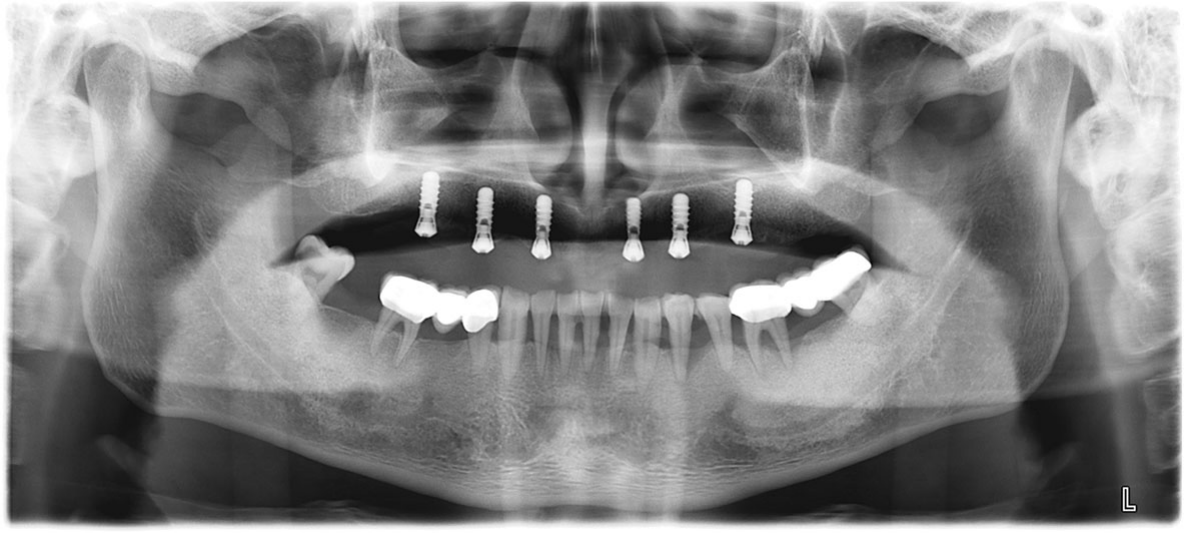
Post-placement panoramic x-ray

Following surgical stent fabrication, six Straumann STL RN implants (Straumann AG, Switzerland) were inserted in the maxilla using a flapless approach (Fig. 5). During the procedure, the surgical guide was firmly stabilized with finger pressure on the palate. After the implant placement, the maxillary denture was relined with Viscogel (Dentsply, USA) (Fig. 6) and delivered back to the patient with instructions on oral hygiene and diet. A post-surgical panoramic x-ray was obtained (Fig. 7).

**Fig. 8:**
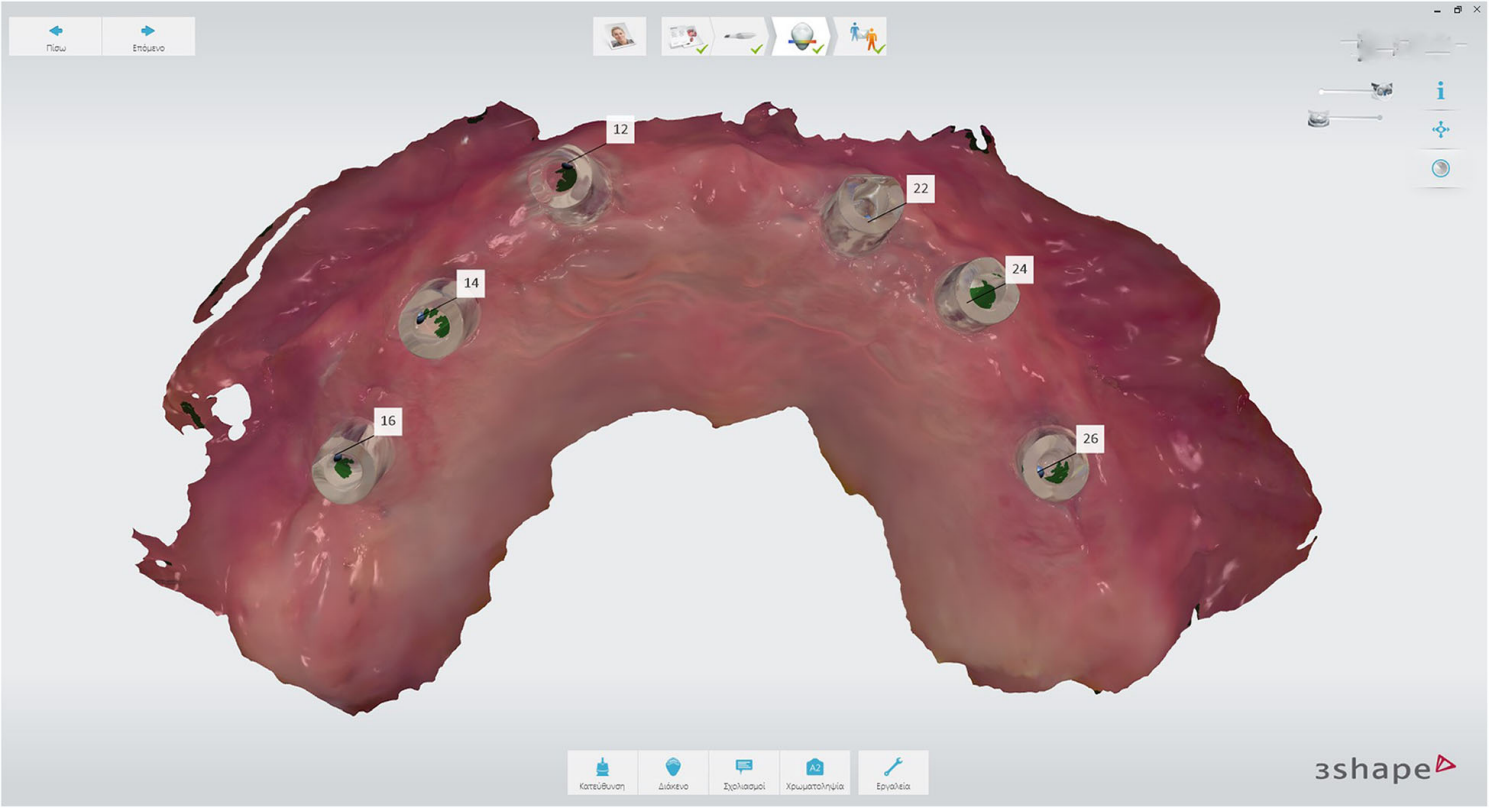
Intraoral maxillary scan

Following 4 months of healing, intraoral scanbodies (Straumann CARES Mono) were hand-tightened on the implants with the angled surfaces facing buccally according to manufacturer’s instructions (Fig. 8) and an intraoral digital impression of the maxillary arch was acquired using an intraoral scanner (Trios3, 3Shape, Denmark) and the official scan strategy as suggested by the manufacturer. A mandibular digital impression was also obtained.

**Fig. 9:**
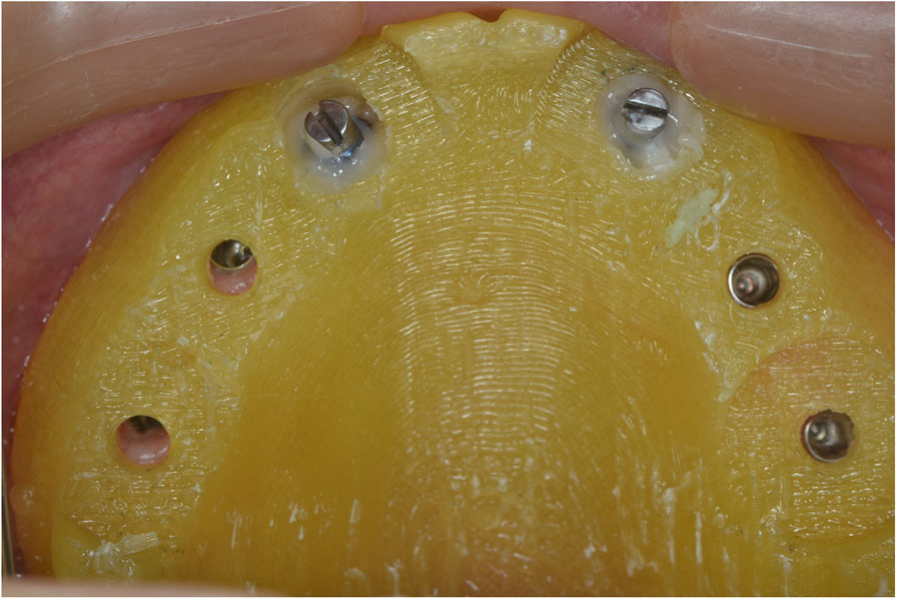
Surgical guide secured onto mesial implants with modified implant carriers showing preserved central incisors and first molars

**Fig. 10:**
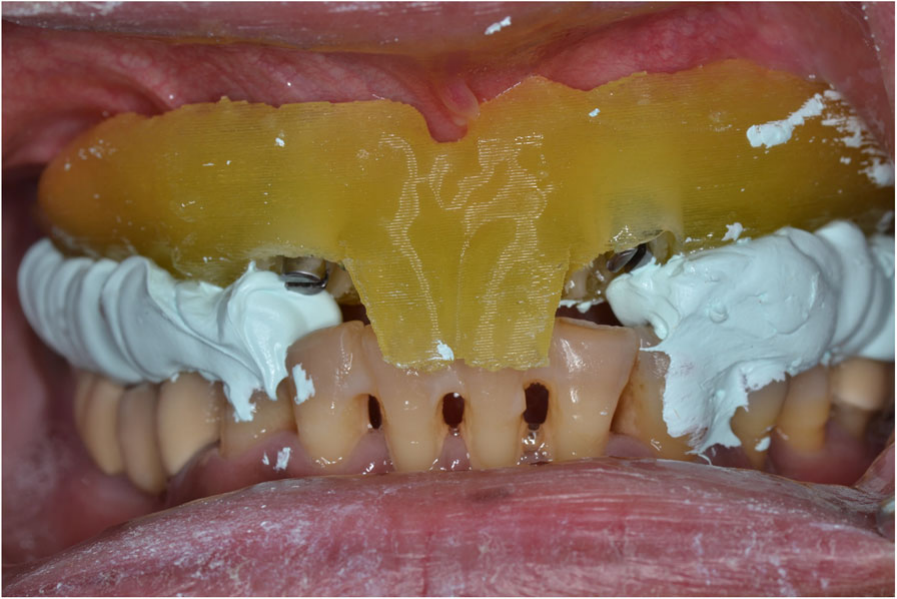
Bite registration material was used to record centric occlusion

To register the OVD already established with the immediate denture, the metal inserts were removed from the stent and the surgical guide was secured on the mesial implants using modified implant carriers and flow resin (Fig. 9). The preserved central incisors on the stent served as an anterior stoppage for the correct anterior-posterior position of the mandible, and the first molars on the stent helped to reproduce the established vertical dimension of occlusion. Although the mesial portion of the molars had been removed from the stent to facilitate implant placement, the distal portion of the teeth was adequate in preserving the correct OVD. To aid in this process, a bite registration material (Prestige Bite, Vanini, Italy) was used to further stabilize the centric relation (CR) at the established OVD (Fig. 10).

**Fig. 11:**
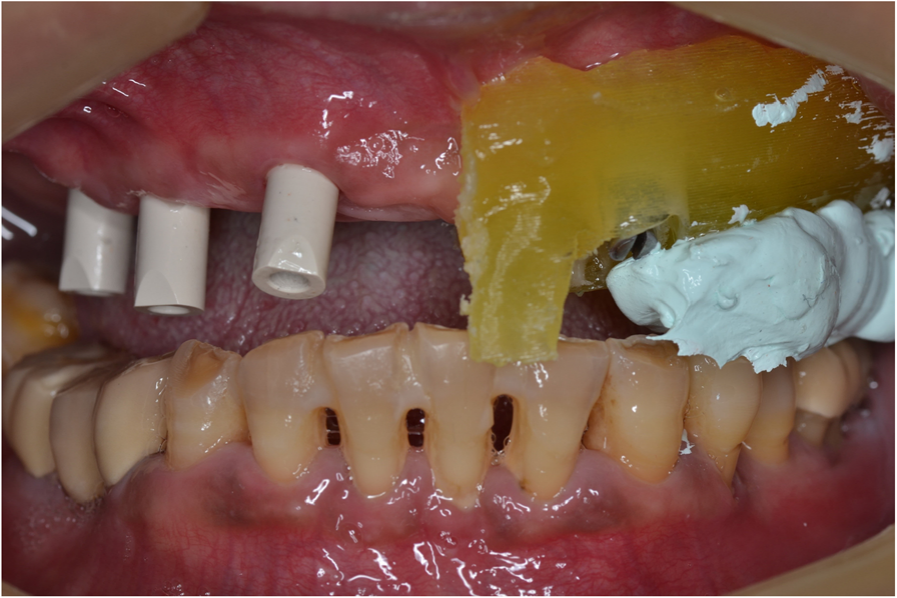
Right hand-side bite registration

**Fig. 12:**
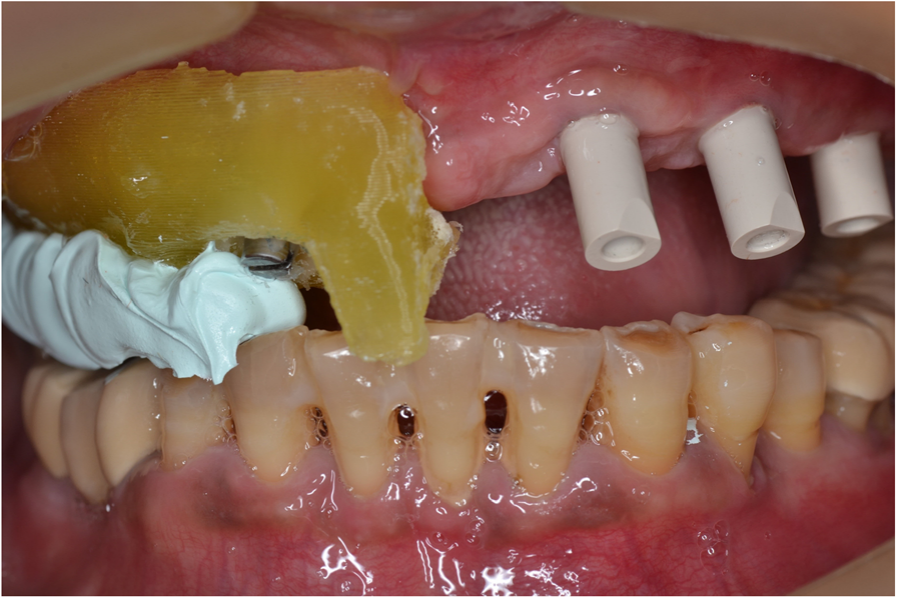
Left hand-side bite registration

**Fig. 13:**
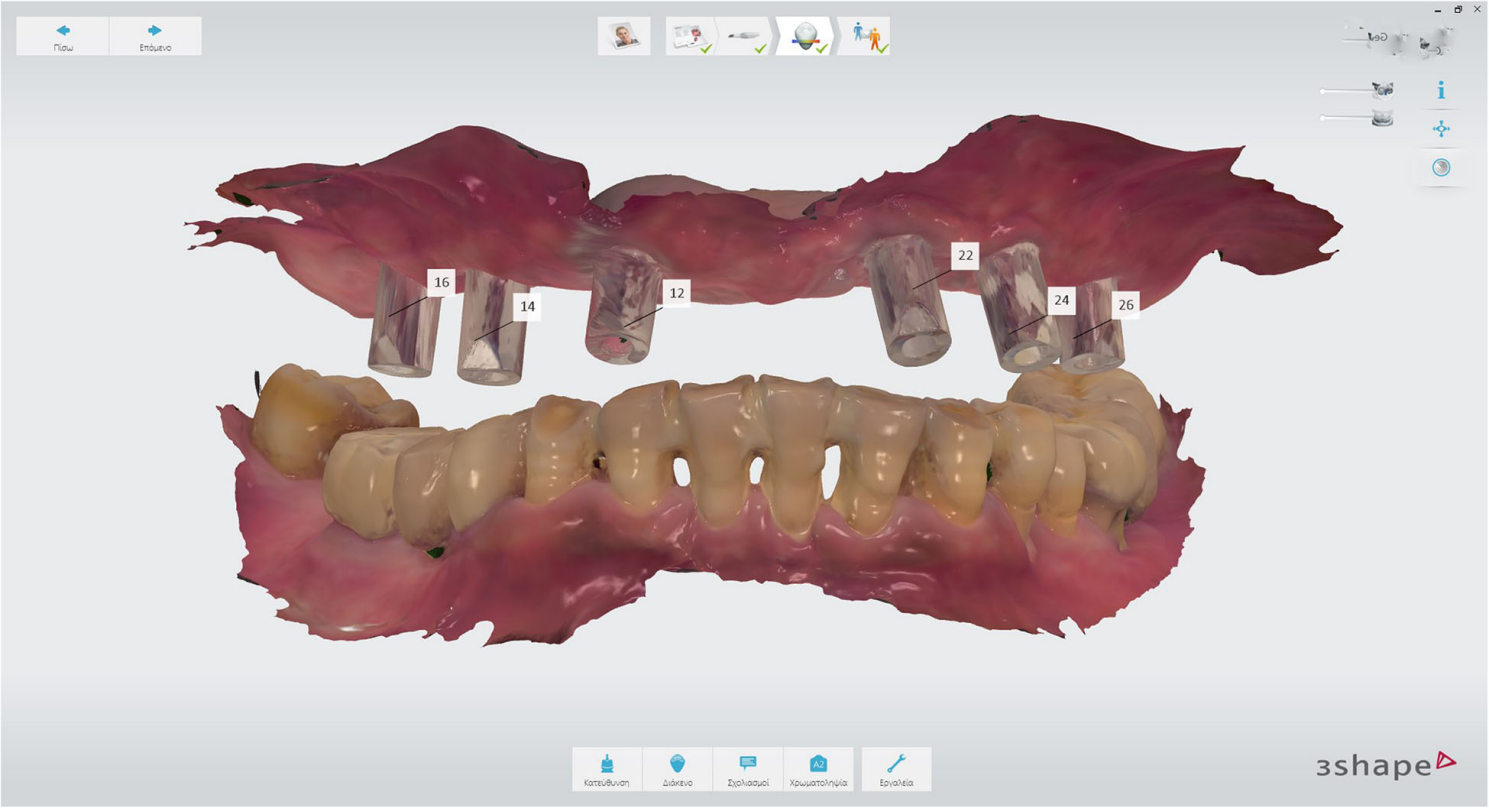
Bite registration finalized

**Fig. 14:**
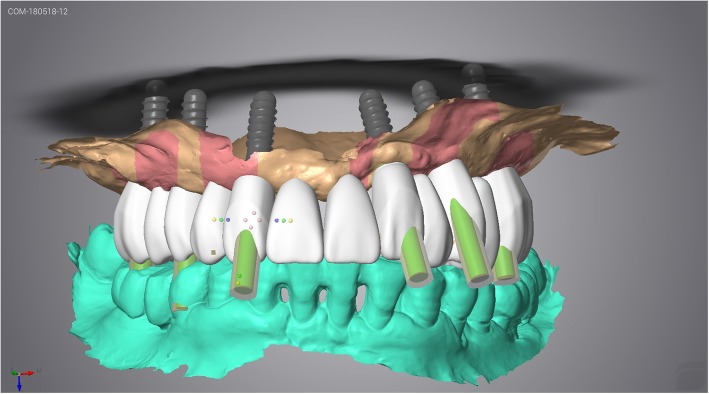
Final restoration designed in CAD software

**Fig. 15:**
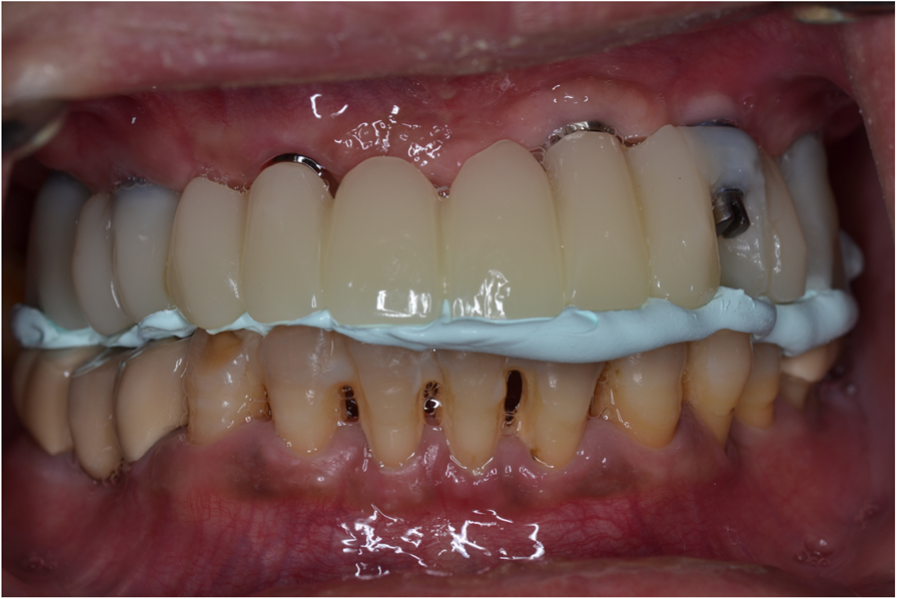
PMMA milled prototype

**Fig. 16:**
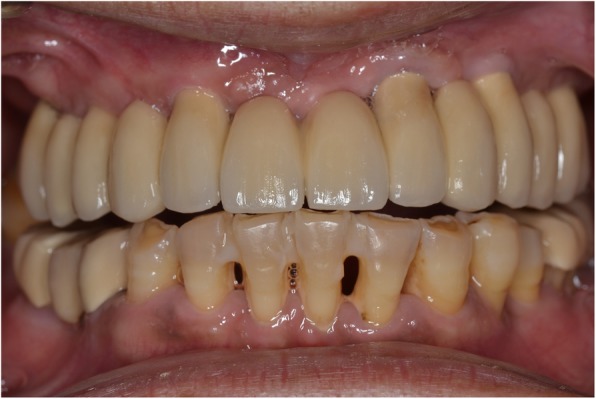
Final prosthesis in situ

Following OVD recording, the guide was sectioned in half along the midline. Each half was firmly kept in place with the aid of the corresponding implant carrier and bite registration material in closure and used interchangeably together with the scanbodies of the contralateral side to register the bite using the intraoral scanner’s bite registration module and the scan strategy suggested by 3Shape (Figs. 11 and 12). The complete digital bite registration was finalized in the IOS software (Fig. 13), and the case was inserted in a CAD design software (Dental Wings, Canada) (Fig. 14). A milled PMMA prototype (Delta Techim, Italy) was constructed and used for the verification of anterior esthetics, and the minor adjustments in the established OVD and CR were finalized using a bite registration material. (Prestige Bite, Vanini, Italy) (Fig. 15). A full-arch cement-retained metal-ceramic restoration on Straumann synOcta abutments (Straumann AG, Switzerland) was finally fitted (Fig. 16).

## Discussion

In the case presented, six endo-osseous implants were installed in the fully edentulous maxilla using a flapless approach with a pilot-guided surgical stent and the patient was restored with an implant-supported fixed prosthesis in a semi-digital approach. Accuracy in the digital registration of the position of the implants and of the OVD was of utmost importance. Hence, an implant-stabilized occlusal rim was deemed necessary.

The surgical guide used during the guided implant placement was modified and served as a precise occlusal rim [24]. Securing the guide onto the implants using the implant carriers and against the opposing teeth by means of the bite registration material served to accurately reposition it in the mouth even after the stent was sectioned in half. Clinically, no mobility of the sectioned stent was observed after fixation to the implant.

Since the implant placement guide was produced digitally from a denture scan, it was an accurate copy of the patient’s existing denture reproducing all important aspects of the prosthesis such as OVD and centric relation (CR). This eliminated the need for an extra appointment for occlusal parameters’ determination and registration [23]. Also, the preserved central incisors relayed useful data regarding tooth size and position to the lab.

Fabrication of an occlusal rim by means of conventional impression taking was not required in our case, leading to less chairside time for the patient. Indeed, reducing treatment time and number of appointments needed leads to less discomfort for the patient and is highly appreciated by both dentists and patients.

## Conclusions

This article presented a method for direct digital bite registration at a predetermined OVD in a patient with fully edentulous maxilla with six implants. The need for conventional impression making to produce an occlusal rim was eliminated. Digital bite registration allowed for a digital workflow, thus ensuring more comfort for the patient. In conjunction with the digital impression taking of the maxilla and mandible, this technique can lead to complete digital workflow encompassing all the merits of this particular workflow.
